# Development and evaluation of a colorimetric LAMP-based biosensor for rapid detection of a nosocomial infection agent, *Citrobacter freundii*

**DOI:** 10.1038/s41598-023-49329-1

**Published:** 2023-12-11

**Authors:** Hamidreza Mollasalehi, Faezeh Esmaili, Dariush Minai-Tehrani

**Affiliations:** Department of Microbiology and Microbial Biotechnology, Faculty of Life Sciences and Biotechnology, Shahid Beheshti University, Velenjak, Tehran, 1983969411 Iran

**Keywords:** DNA and RNA, Diagnostic markers, Bacterial infection, Oligonucleotide probes

## Abstract

Simple and fast diagnosis of *Citrobacter freundii* which is an important cause of nosocomial infection in human is crucial to achieve early treatment. We have developed and evaluated an optical LAMP-based biosensor for the visual detection of *C. freundii* for the first time. The efficiency of the assay was investigated and compared to PCR method. The selectivity and specificity of the biosensor were analyzed using *Morganella morganii*, *Enterobacter aerogenes*, *Pseudomonas aeruginosa*, *Yersinia enterocolitica*, *Shigella sonnei*, *Serratia marcescens*, *Burkholderia cepacia* and *Klebsiella pneumoniae* and a mixed-culture medium. Endpoint analysis using hydroxy naphthol blue was applied, and the color change to sky blue and no color change from violet indicated positive and negative results, respectively. The absorption at 650 nm was measured 0.39 for the positive sample, while the mean absorption of the test samples, including water, was 0.23. The specificity of the method was equal to that of PCR. However, the sensitivity was determined as 12.24 fg/µL of the genomic content of *C. freundii*, higher than PCR assay. The developed LAMP-based method provided a rapid and accurate technique for molecular diagnostics of *C. freundii*, making it a suitable technique for point-of-care diagnostics in cases of urgent situations.

## Introduction

Despite the detection of *Citrobacter freundii* in water, soil, food, and the intestinal tracts of animals and humans, it is able to cause various diseases^1^. Bacteremia^2^, meningitis^3^, peritonitis^4^, nosocomial infections, infections in urinary and respiratory tracts^5^, septic arthritis^6^ as well as healthcare-associated infections^7^, especially in infants are some to mention. The infection is associated with high mortality rate of around 33–48% with *Citrobacter* bacteremia^8^. The majority of the reported cases with *Citrobacter* infection is caused by *C. koseri* and *C. freundii* genomospecies^9,10^. In particular, the high mortality rate of *C. freundii* is due to ineffective antibiotic therapy, since these species encode chromosomal inducible ampC *β*-lactamase genes associated with increased resistance to multiple antibiotics including extended-spectrum cephalosporins^11–13^. Therefore, the diagnosis of the bacteria is in demand.

Rapid diagnosis of *C. freundii* as an infectious agent is vital for early treatment. There are various conventional methods for identifying *C. freundii*, such as culture-based biochemical tests; however, such methods are time-consuming and require a confirmatory test^14–16^. Therefore, molecular methods such as polymerase chain reaction (PCR) need to be developed due to appropriate sensitivity and high speed while they have some limitations namely the need for expensive equipment and specialized personnel^17^. The isothermal amplification techniques were introduced for nucleic acid amplification at a constant temperature using minimum required equipment^18^. The most common isothermal methods are strand displacement amplification (SDA), nucleic acid sequence-based amplification (NASBA), isothermal recombinase polymerase amplification (RPA), helicase-dependent amplification (HDA), multiple displacement amplification (MDA), and loop-mediated isothermal amplification (LAMP)^19^. Moreover, a newly discovered isothermal amplification method is used to detect microorganisms called ladder-shape melting temperature isothermal amplification (LMTIA) that produces nucleic acid at a constant temperature by a thermostable DNA polymerase and one pair of primers or two pairs of nested primers with high specificity and sensitivity^20,21^. Among the diagnostic methods, loop-mediated isothermal amplification (LAMP) is an easy and efficient technique for the detection of microorganisms as an alternative to PCR-based methods^22^.

The LAMP assay is a diagnostic tool based on isothermal amplification. The LAMP method is carried out by a special DNA polymerase with strand displacement activity and employs four or six primers which increases the specificity compared to the PCR-based methods. The LAMP reaction includes two main stages: initiation stage and exponential amplification stage^23^. In brief, a stem-loop structure is produced by four distinct primers with strand displacement feature of the polymerase. The exponential amplification stage continues with self-priming of the stem-loop structure followed by the inner primers attaching to the loop region^18^. Loop primers accelerate the whole process of exponential amplification which enhance creation of cauliflower-like oligonucleotide products^24^. The LAMP product should be visualized and detected. The colorimetric detection methods are suitable candidates to facilitate laborious post-amplification analysis^25^. These approaches utilize indicators including H^+^-sensitive phenol red^26^, metal indicators e.g., hydroxy naphthol blue (HNB)^27^, and fluorescent indicators namely SYBR green I^28^. Furthermore, nanomaterial-based colorimetric detection using Au nanoparticles has been well developed^29^. The colorimetric assays are time-saving capable of one-pot real-time detection with low-cost and less instrumentation.

**Figure 1:**
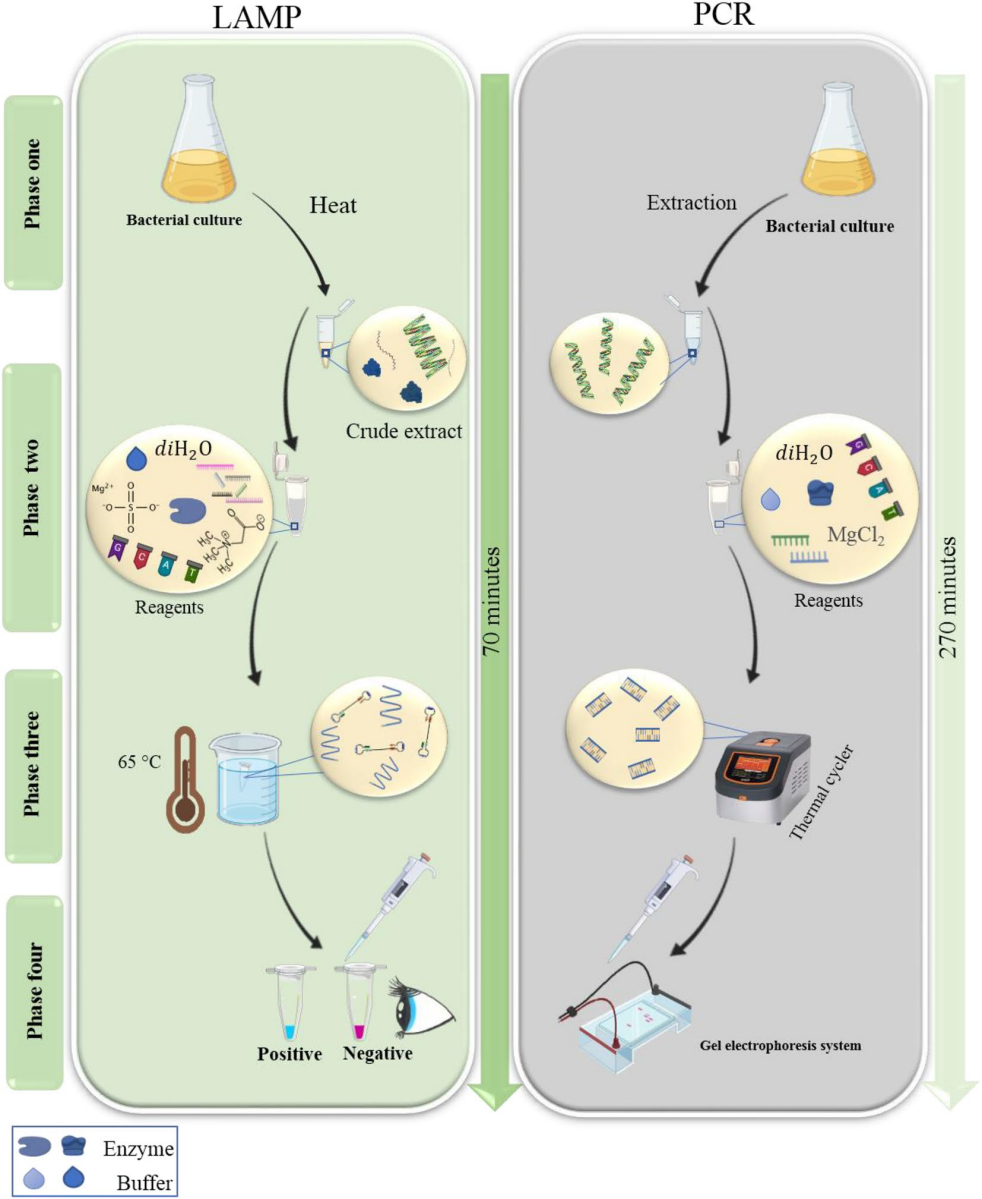
Overview of the current study. Visual LAMP isothermal assay is developed and compared with the conventional gel-based non-isothermal PCR method for the detection of *C. freundii*.

This study intends to develop a LAMP method for the rapid and specific identification of *C. freundii* and also aims to compare its efficiency with the non-isothermal counterpart (PCR). Visualization of LAMP products was performed using a metal indicator sensitive to changes in magnesium concentrations allowing point-of-care analysis with the naked eye. The specificity of the assay was evaluated and the sensitivity plus selectivity were investigated. An overview of the study is presented in the Fig. 1.

## Material and methods

### Chemicals and bacterial culture

All primers were ordered from Metabion, (Germany). The dNTP mixture and enhancer buffer were from Kawsar Biotech, (Iran). The DNA extraction kit, loading dye, *Taq* DNA polymerase, PCR buffer, magnesium chloride, and molecular-weight size marker were from Cinnaclone Co., (Iran). Stain was from DeNA Gene Tajhiz, (Iran). Agarose was from Gene Fanavaran co. (Iran). *Bst* 2.0 warmstart DNA polymerase, isothermal amplification buffer, and magnesium sulfate were obtained from New England Biolabs (UK). Tryptic soy broth (TSB) and tryptic soy agar (TSA) were from Liofilchem, (Italy). Tris-base was obtained from Biochem Chemopharma, (France). Boric acid, ethylene diamine tetra acetic acid, and betaine were purchased from Merck (Germany). All chemicals were of molecular biology grade. Bacterial samples were purchased from Iranian Research Organization for Science and Technology (IROST), (Iran). Lyophilized bacteria of C. *freundii* and eight other species were revived in 100 mL of TSB culture medium at 37 °C for 18–24 h. Moreover, before molecular detection testing, purity and probable contamination were examined with bacterial culture in TSA, and then DNA extraction was performed on the liquid culture media.

### Nucleic acid extraction

The genomic DNA was extracted using a gram-negative DNA extraction kit and the boiling method. According to the protocol of the kit prescribed by the manufacturer, briefly, 10–20 mg of cultured bacterial cells were collected by centrifugation at 2000*g*. The collected cells were incubated with both prelysis buffer and ribonuclease A at 37 °C for 15 min. Finally, Proteinase K followed by lysis buffer were added and the washing steps were applied three times. Pure genomic DNA was used for downstream procedures. In the boiling method, the bacterial cells were collected by centrifugation at 2000*g* and washed once using PBS 0.9% then they were heated in boiling water for 10 min. After centrifugation at 2000*g* for 5 min, the supernatant containing nucleic acid was utilized for further analysis. The quality and quantity of extracted DNA were examined using spectrophotometry at 260/280 nm and gel-electrophoresis methods. All samples were analyzed under identical situations for running the test.

### Amplification and primer design

Various genes in the genome of *C. freundii* were investigated for specificity using BLAST tool at NCBI. A distinct sequence of *ydcF* gene coding Ydc family protein with accession number EGX89_RS01790 was selected in *C. freundii* gene that varies in other related bacteria. Six primers, including two inner primers, two outer primers, and two loop primers, were designed by the LAMP Designer software 1.01 (Table 1) to amplify the specific target sequence. The LAMP reaction was performed in 12.5 μL mixture containing 10X isothermal amplification buffer (1.25 μL), 6 mM MgSO_4_ (0.75 μL), 1.5 M betaine (3.75 μL), 1.4 mM of each dNTP (1.75 μL of dNTP mix), 0.5 μL of 25X primer mix including 40 μM FIP and BIP primers, 5 μM F3 and B3 primers, 10 μM LB and LF primers, 0.5 μL of extracted nucleic acids and 4U *bst* DNA polymerase. The LAMP mixture was then incubated at 65 °C for 60 min to amplify the target sequence, followed by a 2 min incubation stage at 80 °C to inactivate the enzyme. PCR mixture was prepared in total volume of 50 μL containing 10× PCR buffer (5 μL), MgCl_2_ (1.25 mM), F3 primer (0.25μM), B3 primer (0.25 μM), dNTP mix (0.32 mM), Taq DNA polymerase (2U), 1 μL DNA template, and enhancer buffer (1.5 M). Thermocycling conditions included 95 °C for 5 min, followed by 35 cycles of 95 °C for 30 s, 54 °C for 30 s and 72 °C for 30 s, with the final elongation step at 72 °C for 5 min. The LAMP amplicons were evaluated using visual detection of HNB followed by spectrophotometric analysis of visible spectra while the PCR products were analyzed by gel electrophoresis.

**Table 1:** The designed LAMP and PCR primers for detection of *Citrobacter freundii*.

Method	Name	Sequence (5′⟶3′)	Length	Reference
LAMP	F3	AGATTGCGTGATTCTGGC	18	This study
	B3	TGTTGGTGGACTGATCCT	18	This study
	FIP	TGGCAGCATACAGAAAGGTCGGGTAGCGAAAGAGCAACA	39	This study
	BIP	CGCAGAGCATCCACGCTAGATATCGGCAAGGATCGC	36	This study
	LF	GATACCACCGCTAATCAGTAGG	22	This study
	LB	ACACGATTCGTACCACCG	18	This study
PCR	F3	AGATTGCGTGATTCTGGC	18	This study
	B3	TGTTGGTGGACTGATCCT	18	This study

### Specificity, sensitivity and selectivity evaluation of the assay

The specificity of assay was analyzed by using the genomic DNA of nine bacteria from different genera and species, including *C. freundii* (Persian Type Culture Collection 1600), *Morganella morganii* (PTCC 1078), *Enterobacter aerogenes* (PTCC 1221), *Pseudomonas aeruginosa* (American Type Culture Collection *9027*), *Yersinia enterocolitica* (PTCC 1151), *Shigella sonnei* (PTCC 1777), *Serratia marcescens* (PTCC 1621),* Burkholderia cepacia* (ATCC *25416*), *and Klebsiella pneumoniae* (PTCC 1053). In addition, the sensitivity was investigated using decimal serially diluted DNA templates of *C. freundii* from 10^–1^ to 10^–8^. Mixed microbial cultures were used to evaluate the selectivity of the method. Identical volume of all different bacteria used in this study were inoculated into a culture medium and incubated overnight at 37 °C. Positive control contained *C. freundii* together with all other bacteria, while negative control sample lacked *C. freundii*. DNA extraction was performed as explained in “Nucleic acid extraction” section.

## Results

We aimed to develop a colorimetric LAMP assay for *C. freundii* detection and analyze its performance over PCR method. In that regard, the *ydc F* gene coding Ydc family protein was selected as target biomarker.

### Analysis of genomic nucleic acid

**Figure 2:**
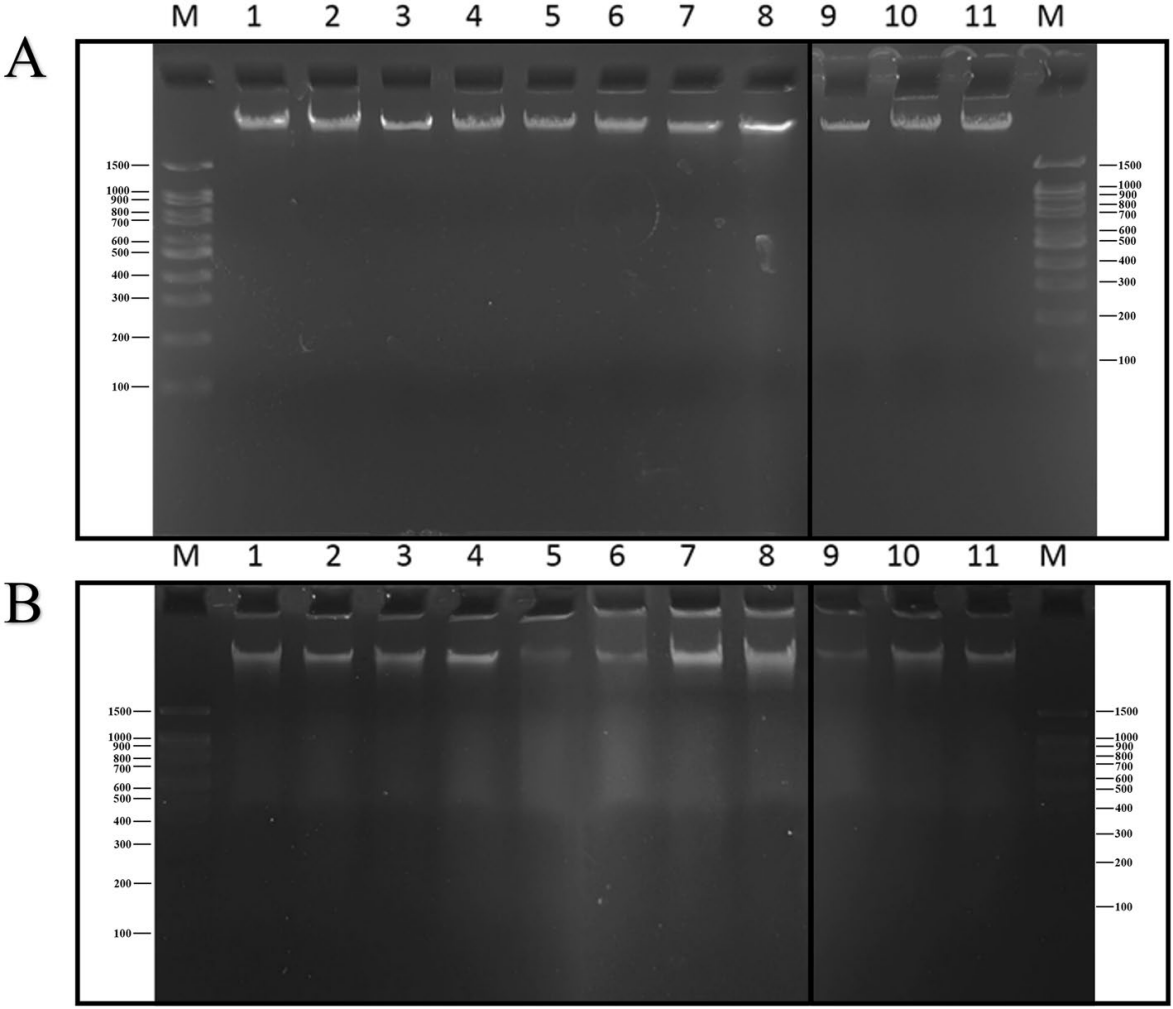
Evaluation of the quality of the extracted genomic nucleic acid on 1% agarose gel. (**A**) The extracted genomic DNA using extraction kit (**B**) extracted genomic DNA using boiling method. M: DNA size marker; Lane1: *C. freundii*; Lane2: *M. morganii*; Lane3: *B. cepacia*; Lane4: *S. marcescens*; Lane5: *Y. enterocolitica*; Lane6:* S. sonnei*; Lane7: *E. aerogenes*; Lane8:* P. aeruginosa*; Lane9:* K. pneumoniae*; Lane10: positive mixed culture; Lane11: negative mixed culture.

The ratio of A260/ A280 nm was measured for all tested bacteria and presented in the supplementary material. The absorbance ranging between 1.06 to 2.08 was recorded and values above 1 was considered of appropriate purity for further analysis. Moreover, the DNA concentration was measured for all extracted nucleic acids which ranged between 22 to 186 ng/μL. In addition, the result was confirmed by a high-molecular-weighted band of genomic DNA on gel-electrophoresis (Fig. 2).

### Specificity analysis of the methods

**Figure 3:**
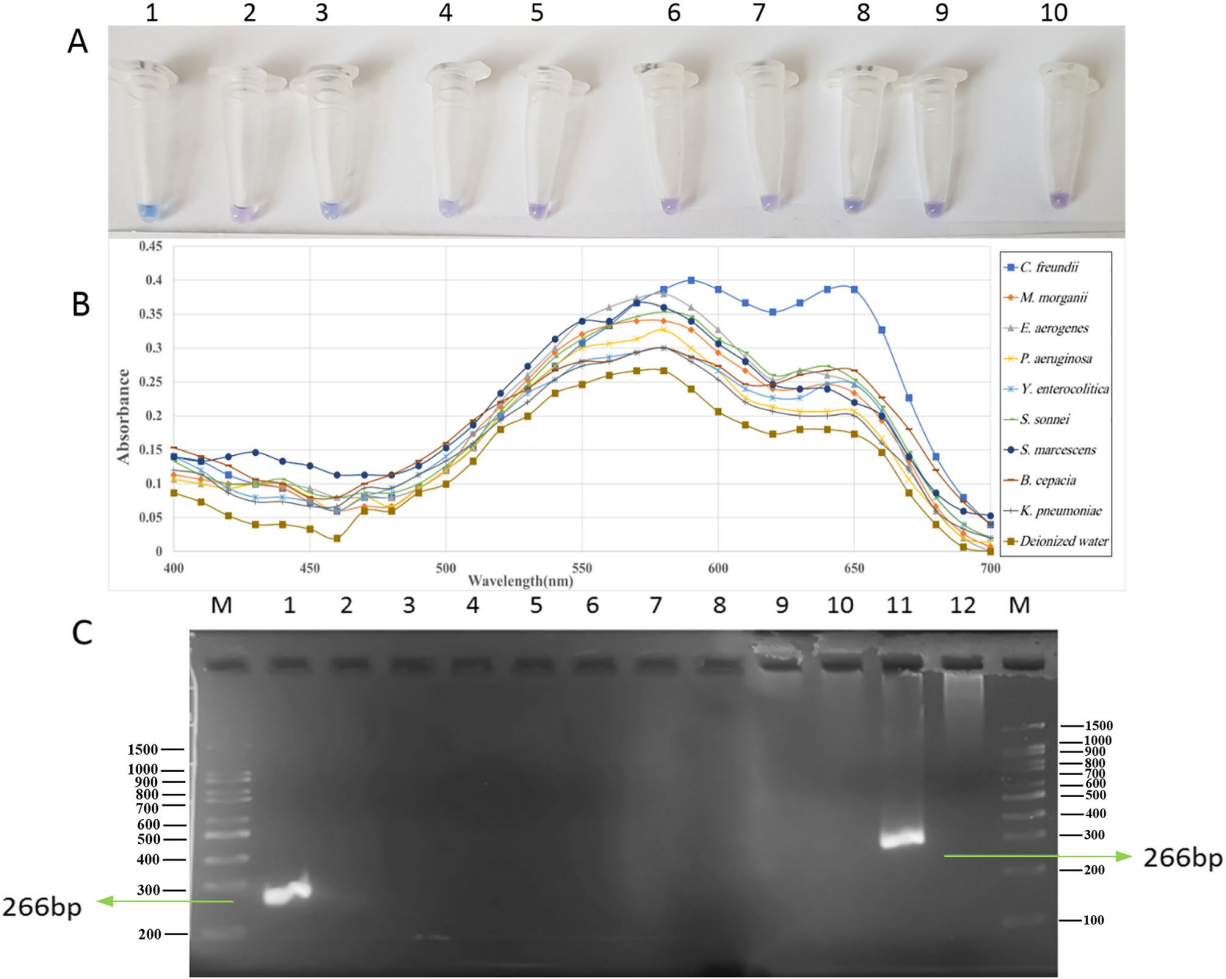
Specificity analysis of detection method. (**A**) Evaluation of LAMP products using colorimetric method 1: *C. freundii*, 2: *M. morganii*, 3: *E. aerogenes*, 4: *P. aeruginosa,* 5: *Y. enterocolitica*, 6: *S. sonnei*, 7:* S. marcescens*, 8:* B. cepacia*, 9:* K. pneumoniae*, 10: Deionized water (without DNA template). (**B**) Quantitative evaluation of the colorimetric method. Visible spectrophotometric analysis between 400 and 700 nm was recorded. (**C**) Agarose gel (2%) electrophoresis of PCR products. M: DNA size marker, Lane1: *C. freundii*, Lane2: nuclease free water, Lane3: *P. aeruginosa*, Lane4: *B. cepacia*, Lane5: *Y. enterocolitica*, Lane6: *K. pneumoniae*, Lane7: *M. morganii*, Lane8: *E. aerogenes*, Lane9: *S. sonnei*, Lane10: *S. marcescens*, Lane11: positive mixed culture, Lane12: Negative mixed culture.

The specificity of the developed colorimetric LAMP method was investigated using various bacteria from related species to *C. freundii*. In this assay, endpoint approach was used in which color changing from violet to sky blue was considered as positive while no color change retaining violet was considered as a negative result. It was shown that C. *freundii* sample changed to sky blue whereas other tested bacterial samples remained violet (Fig. 3A). Furthermore, the quantitative evaluation in the visible spectrum (400–700 nm) by spectrophotometry method was performed. As it is shown in Fig. 3B, two peaks were recorded at 590 and 650 nm for *C. freundii* sample with the intensity of 0.4 and 0.39, respectively. However, the 650 nm peak presented more distinct variation in comparison with other bacteria and was selected as an appropriate wavelength indicator. The difference between the maximum (positive sample) and minimum (negative control) peaks was recorded 0.21. Moreover, the specificity of PCR assay was also examined. A 266 bp band on the agarose gel electrophoresis was obtained for C. *freundii* sample. On the other hand, no amplicon band was detectable on the gel for the other tested samples (Fig. 3C).

### Sensitivity analysis of the methods

**Figure 4:**
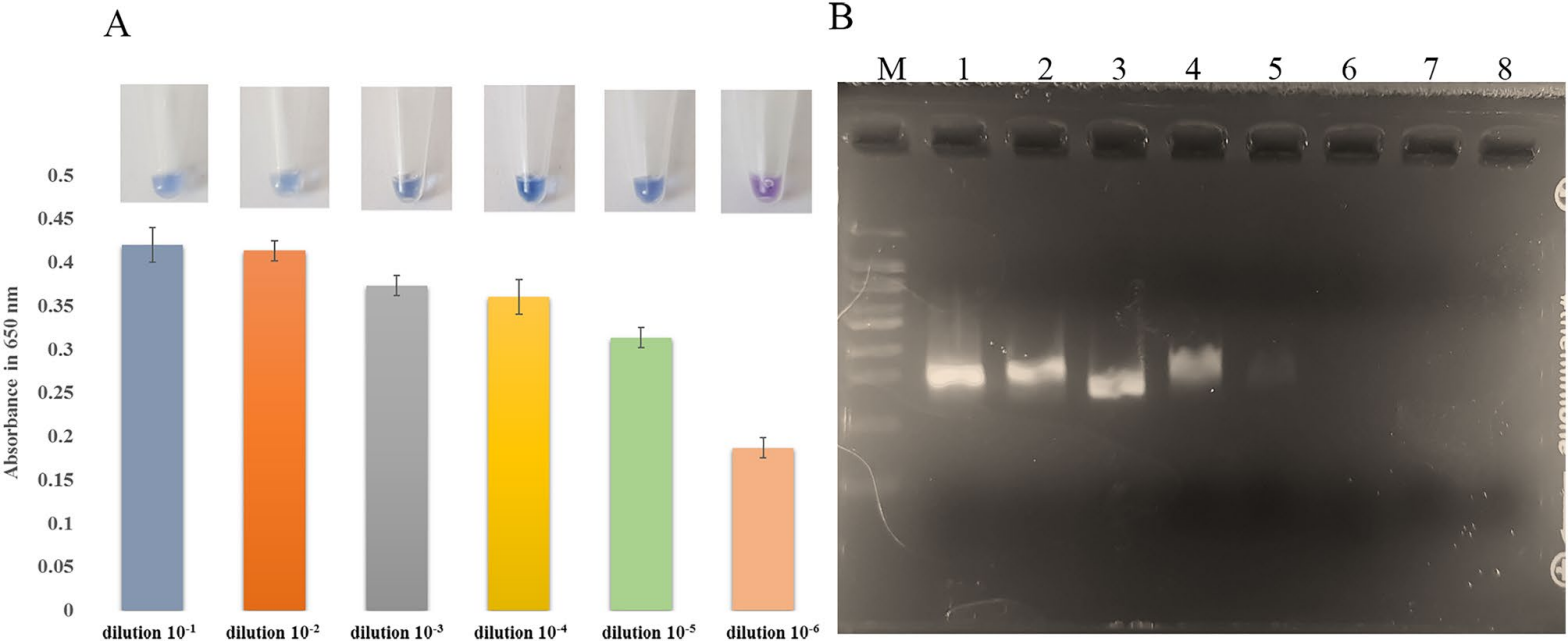
Sensitivity evaluation of developed method. (**A**) quantitative evaluation of colorimetric LAMP products by spectroscopic evaluation at 650 nm. (**B**) Agarose gel (2%) electrophoresis of PCR products to evaluate the sensitivity Lane M: DNA size marker; Lane 1: dilution 10^–1^, Lane 2: 10^–2^, Lane 3: 10^–3^, Lane 4: 10^–4^, Lane 5: 10^–5^, Lane 6: 10^–6^, Lane 7: 10^–7^, Lane 8: 10^–8^.

The sensitivity of the designed colorimetric LAMP assay and PCR method was analyzed using *C. freundii* as template. As shown in Fig. 4A, the color change in the LAMP assay was observed up to the dilution of 10^–5^, which equals approximately 12.24 fg/µL (original concentration, 30.6 ${\text{ng}}/\mathrm{\mu L}$, dilution factor, ${10}^{-5}$, volume of 0.5 $\mathrm{\mu L}$ to final volume of 12.5 $\mathrm{\mu L}$). Quantitative evaluation of colorimetric detection at 650 nm was performed and the range of 0.42 to 0.31 for dilutions 10^–1^ to 10^–5^ was attained, respectively. It should be noted that the absorbance of 0.18 for dilution 10^–6^ was considered as negative result (Fig. 4A). Also, the PCR sensitivity results showed the 266 bp band of the desired sequence up to the dilution of 10^−5^ which equals approximately 11.76 fg/µL while no amplicon band was detectable for the 10^–6^ dilution (original concentration, 58.8 ${\text{ng}}/\mathrm{\mu L}$, dilution factor, ${10}^{-5}$ volume of 1 $\mathrm{\mu L}$ to final volume of 50 $\mathrm{\mu L}$) (Fig. 4B).

### Selectivity evaluation of the method

**Figure 5:**
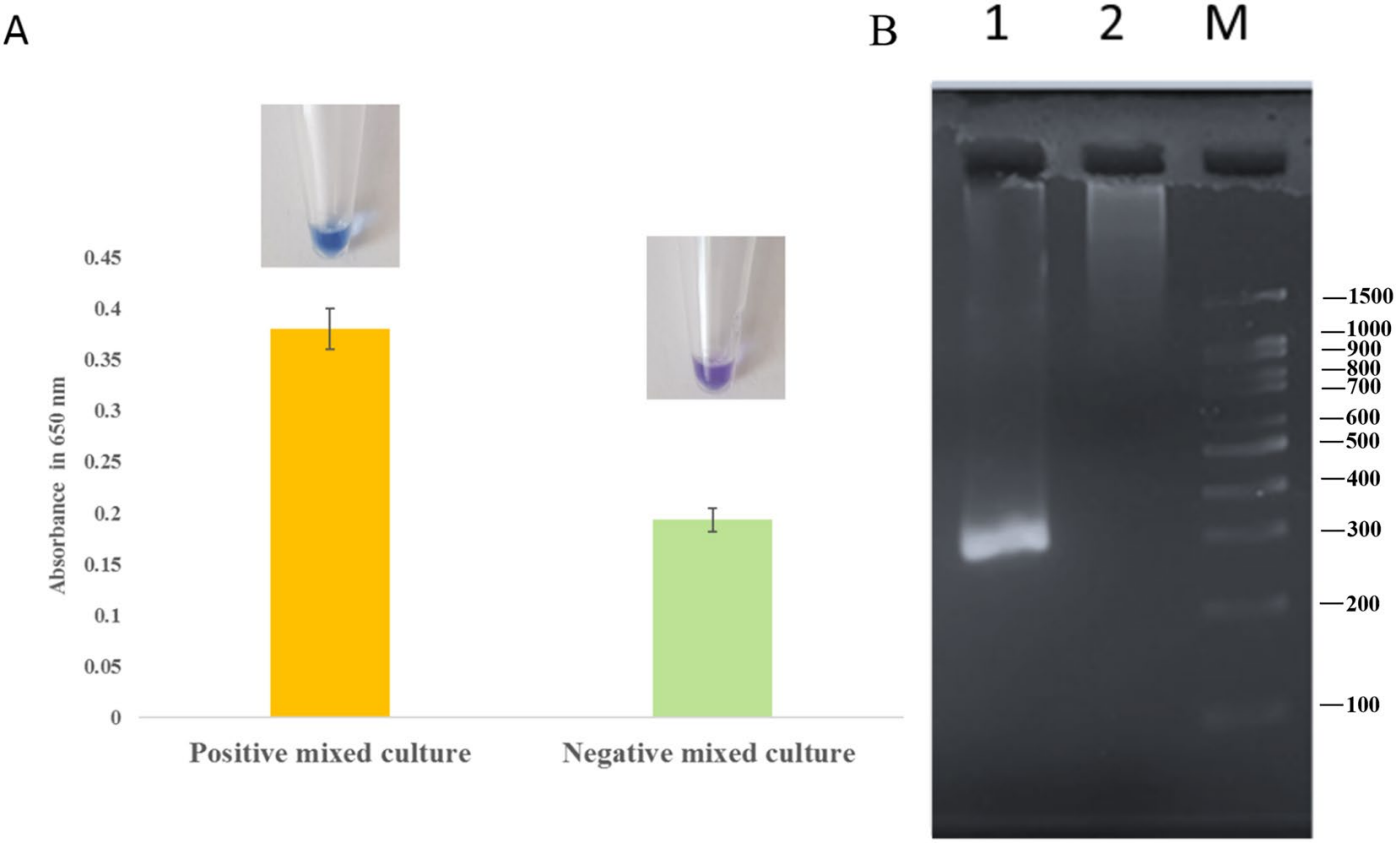
Selectivity analysis of developed LAMP and PCR assay. (**A**) Selectivity evaluation of LAMP using visual and spectroscopic assay. (**B**) Agarose gel (2%) electrophoresis of PCR products of mixed cultures. Lane M: DNA size marker; Lane1: Mixed culture PCR products containing all bacteria; Lane2: Mixed culture PCR products containing all bacteria except *C. freundii.*

The selectivity of the developed LAMP and PCR methods were investigated by extracted genomic DNA of mixed bacterial cultures. The LAMP assay presented a color change for the positive mixed-culture with no change for the negative mixed-culture (Fig. 5A). Moreover, the absorbance measurement at 650 nm was 0.38 for the positive sample and 0.19 for the negative sample (Fig. 5A). The PCR Selectivity result indicated a significant band for positive mixed culture samples and no amplicon band for the negative mixed culture (Fig. 5B).

## Discussion

Prompt and accurate identification of *C. freundii* is essential for effective implementation of infection control approaches due to its pathogenicity in infants and adults. To date, *C. freundii* detection has been performed using currently available method, such as biochemical tests, culture-based methods, and PCR-based assays^14,16,30,31^. However, they need a long hands-on time leading to some inaccurate results, high costs, and high-tech facilities. Therefore, an accurate, fast identification method is required. In this study, two detection methods, LAMP and PCR, have been evaluated in terms of *C. freundii* identification and colorimetric LAMP outperforms PCR in terms of simplicity, cost, time, and being user-friendly. To the best of our knowledge, this is the first report on the specific Identification of *C. freundii* using a colorimetric isothermal amplification assay. The developed method could identify the target bacterium using the *ydcF* gene as a nucleobiomarker in only one hour at a constant temperature.

The LAMP amplification identification is carried out by various methods including non-specific identification e.g., using fluorescent intercalating dyes^32^ and specific detection comprising probe-based approaches^33^. dNTPs polymerization in the LAMP amplification system results in high concentrations of pyrophosphate ions which react with the Mg^2+^ ions to produce magnesium pyrophosphate. The change in Mg^2+^concentration was visualized by HNB as a metal indicator^27^. The indicator benefits remarkable advantages of simplicity, safety, and affordability without any requirement for excitation. The mentioned wavelength was selected as the indicator for quantitative evaluation. In a study on HNB to identify LAMP products, a scan of the spectrum was performed and an identical wavelength was selected using λ DNA as target^27^. In another study on influenza H1N1 virus utilizing the RT-LAMP method the same wavelength was selected^34^.

The specificity of the developed method for the detection of *C. freundii* was investigated. In a study using conventional method including biochemical test and staining, *C. freundii* was misidentified with *Klebsiella* sp. and *E. coli*^35^. Moreover, in a PCR-based study *C. freundii* was identified by targeting the *cfa* gene, which was present in both *C. freundii* and *Citrobacter braakii*^30^. Meanwhile, in this study, the developed colorimetric LAMP method displayed no cross-reaction among other bacteria. The same primer set was used in the gold-standard PCR assay for validation of the results and the specificity was confirmed.

The limit of detection for the developed PCR and LAMP methods was evaluated. The sensitivity results of the LAMP assay indicated the LOD of femtogram per microliter scale. Compared to other diagnostic LAMP studies on *Salmonella *sp.^36^ and *Streptococcus agalactiae*^37^ with the LOD of picogram per microliter scale and Botrytis cinerea^38^ with a LOD of nanogram per microliter scale, higher sensitivity was accomplished. The LOD of the developed PCR assay was almost equal to the LAMP method, however, it should be noted that the input DNA used in LAMP assay was crude extract of the whole cell while the template in PCR assay was purified using a DNA extraction kit. Therefore, the sensitivity of the developed LAMP assay is estimated multiple higher than PCR method. Moreover, the LAMP assay is less sensitive to the environmental reagents in the reaction mixture and also high measured sensitivity was achievable by unequipped eyes and visual detection. This could well guarantee the point-of-care and clinical application of the method.

The capability of a detection method to detect the target among several pathogens similar to clinical samples is of particular importance. In that regard, the selectivity of the LAMP assay was investigated and *C. freundii* in the modified environment among other bacteria was isolated. The mixed microbial culture simulates the clinical situation. According to the same absorbance intensity obtained in the new environment, the developed colorimetric LAMP method indicated acceptable reproducibility with the tested samples (it should be noted that each test was run at least three times). In a study on using LAMP method to detect *Chlamydia trachomatis* the selectivity was also determined using clinical samples^39^. Besides, the selectivity of the developed PCR assay was also evaluated, and the *C. freundii* was detected accurately among the tested samples. Consequently, the designed LAMP and PCR assays indicated equal selectivity results, while the higher sensitivity of the LAMP assay could result in more selectivity in comparison with the PCR assay. The selective feature of the developed method could provide a step forward in designing a diagnostic kit for clinical applications.

The colorimetric LAMP assay includes many advantages over the PCR assay. The colorimetric LAMP amplification performed in simple water bath and visually detected in 70 min while the PCR method required a costly thermocycler and electrophoresis system which was carried out in 270 min^28^. Besides innate simplicity, higher speed, and lower cost, together with the achieved specificity, sensitivity and selectivity, the developed colorimetric LAMP assay could well serve as a suitable technique for detecting *C. freundii* pathogen in more complex environmental systems. The developed method contains multiple innovations as a diagnostic method for the detection of *C. freundii*. Initially, the target gene was used for the first time for detection purposes, and it was completely specific for the target pathogen. Also, the LAMP assay was developed for the first time as a rapid detection method to spot *C. freundii* with high sensitivity and specificity. Moreover, integrating the LAMP assay with an optical biosensing method could increase the applicability and technology readiness (TRL) level of the method. Accordingly, the use of a mixed culture for evaluating selectivity simulated a clinical sample and could be a step forward for future advancement to setting up a kit for clinical determinations. The only limitation of the method would be the lack of a sequence-specific probe for a more reliable detection of the amplified products.

## Conclusion

The developed colorimetric LAMP facilitated selective *C. freundii* detection with high sensitivity and specificity. The designed colorimetric LAMP could be used in situations when straightforward, rapid, and accurate identification of *C. freundii* is needed due to the emergence of the bacteria in infectious outbreaks and resource-limited circumstance.

### Supplementary Information

Supplementary Information.

## Data Availability

All data generated or analyzed during this study are included in this published article (and its supplementary information files).
