# Supplementary material for: Development and evaluation of a colorimetric LAMP-based biosensor for rapid detection of a nosocomial infection agent, Citrobacter freundii
Source: Sci Rep. 2023 Dec 11;13:21896. doi: 10.1038/s41598-023-49329-1 (PMC10713557; doi:10.1038/s41598-023-49329-1)
Supplement: Supplementary file 1 — Supplementary Information. [file 41598_2023_49329_MOESM1_ESM.docx]

**Development and evaluation of a colorimetric LAMP-based biosensor for rapid detection of a nosocomial infection agent, *Citrobacter freundii***

Hamidreza Mollasalehi ^*1^, Faezeh Esmaili^1^, Dariush Minai-Tehrani^1^

Department of Microbiology and Microbial Biotechnology, Faculty of Life Sciences and Biotechnology, Shahid Beheshti University, Tehran, Iran. Postal Code: 1983969411

**Supplementary data**

The following table is related to section 3.1. The table represented optical density and concentration of extracted DNA by DNA extraction kit and boiling methods.

| Bacteria | Boiling | | Kit | |
| --- | --- | --- | --- | --- |
|  | 260nm/280nm | Concentration (ng/*µ*L) | 260nm/280nm | Concentration (ng/*µ*L) |
| *Citrobacter freundii* | 1.23 | 30.6 | 2.01 | 58.8 |
| *Morganella morganii* | 1.16 | 47.04 | 1.89 | 51.7 |
| *Enterobacter aerogenes* | 1.32 | 59.86 | 2.08 | 39.4 |
| *Pseudomonas aeruginosa* | 1.06 | 76.32 | 1.87 | 60.5 |
| *Yersinia enterocolitica* | 1.25 | 22.5 | 2 | 49.1 |
| *Serratia marcescens* | 1.27 | 53.86 | 1.92 | 84.1 |
| *Klebsiella pneumoniae* | 1.28 | 25.24 | 1.90 | 66.9 |
| *Burkholderia cepacia* | 2.01 | 46.28 | 1.79 | 126.9 |
| *Shigella sonnei* | 1.23 | 32.3 | 1.98 | 56.7 |
| Negative mixed culture | 1.12 | 56.42 | 1.82 | 150.3 |
| Positive mixed culture | 1.28 | 64.18 | 1.93 | 185.2 |
